# The influence of motor expertise on gender difference in adolescents’ object-based and egocentric mental rotation ability

**DOI:** 10.1186/s40359-026-04099-z

**Published:** 2026-02-16

**Authors:** Tian Feng, Youxin Wei, Manqi Liang, Yawei Li

**Affiliations:** 1 Department of Physical Education, Henan Sport University, Zhengzhou, Henan China; 2 School of Physical Education, Henan University, Kaifeng, Henan China; 3 School of Kinesiology and Physical Education, Zhengzhou University, Zhengzhou, Henan China

**Keywords:** Mental rotation, Gender, Athletes, Adolescent, Transformation

## Abstract

**Background:**

Gender differences in mental rotation are well established. However, conflicting results were reported when gender was shown to interact with sport expertise and different task transformations in adolescents’ mental rotation.

**Methods:**

Forty-four adolescent subjects (22 divers and 22 nonathletes) participated in the experiment. A mental body rotation task with object-based and egocentric transformation conditions was conducted, and the reaction time, accuracy, and stage performance were recorded.

**Results:**

The results showed that in the object-based task involving cube images, the divers had shorter reaction times than did nonathletes, and the perception speed of athletic boys was faster than that of athletic girls. In the object-based task with body image, athletes’ advantage was confirmed, and the accuracy for girls was significantly greater than that for boys. No gender difference was detected in the egocentric task.

**Conclusions:**

The mental rotation ability of adolescents was found to be significantly influenced by their mental rotation representation and motor expertise, which differed by gender.

**Supplementary Information:**

The online version contains supplementary material available at 10.1186/s40359-026-04099-z.

## Introduction

Mental rotation is an important spatial cognitive ability. In the sports field, the technical and tactical execution of most sports requires athletes to perceive, encode and convert spatial information. It is an imaginary process in which an individual uses transformation to perform two-dimensional or three-dimensional rotations on objects in the mind. According to the classical view, mental rotation includes two different transformations, object-based transformations and egocentric transformations [1], which widely exist in daily life and are influenced by the experience of individuals operating and transforming themselves or objects [2].

In object-based representation, the subjects operated on the objects in the imaginary space from the third-person perspective (objective reference frame). For example, basketball players always need to gather around the coach to receive tactical guidance during the interval in the game. Those players standing facing the coach can only read the tactical board in the coach’s hand upside down, which is an object-based representation. In this representation, the observer fixes his own position and manipulates the object to perform transformation. In the egocentric representation, the subjects imagined themselves transforming in the space from the first-person perspective (subjective frame of reference). Under this representation, the object remains fixed, and the observer establishes a relationship with the object by changing its angle or orientation. In previous studies, egocentric mental rotation was considered to be closely related to body movements. Different task stimuli were also used in these two transformations: egocentric representation is assessed using pictures or schematics related to the human body (e.g., hands, feet, or full human figures), whereas object-based representation is measured using two- or three-dimensional block figures. In addition, the mental rotation information processing stage is divided into three sequential processing stages, namely, the perception stage, the rotation stage and the decision stage [3–5].

From the perspective of the development of mental rotation, somersaults and turns in gymnastics and diving and turns in basketball and football are all performed by the body rotating around a certain axis; therefore, rotation is one of the important technical movements in sports. Studies in gymnastics, football, wrestling, and orienteering found that athletes scored significantly higher than nonathletes in mental rotation tasks involving 3D abstract figures and human figures, and the mental rotation scores of expert athletes were significantly greater than those of novice athletes in the same sport [6–8]. In addition, studies using college students with different majors revealed that the mental rotation ability of students majoring in physical education was significantly greater than that of students with other majors. This result is related to the course study and sports training of physical education majors [9]. However, some studies have reported inconsistent results. Schmit et al. reported no difference in mental rotation speed or mental rotation scores between college running club members and students with no exercise habits [8]. They believe that this result is due to the lower demand for rotation representation in sports, such as running. Therefore, research attention has increasingly focused on examining domain-specific differences in mental rotation performance among athletes participating in distinct sporting activities.

Engaging in diverse sports activities may exert distinct impacts on the development of mental rotation ability. Compared with players in closed sports such as running, players in open sports such as football and basketball need to analyze the opponent’s movement direction and the ball’s trajectory on the spot because this process involves more complex and changeable spatial factors. Therefore, researchers believe that the mental rotation ability of sport participants with more complex spatial information should be better than that of participants with simple spatial information. Moreau et al. compared fencing, judo, and wrestling athletes with running athletes and reported that the former have greater mental rotation ability because, in confrontational sports, athletes need to use different strategies to cope with various complex competition environments [7]. Another study revealed that the mental rotation ability of gymnastics and orienteering participants was better than that of football and running participants [6, 8]. Accordingly, the researchers further proposed that mental rotation performance depends on the compatibility among the physical rotations experienced during physical activity and some components of the mental rotation test, showing the characteristics of “selective influence”. They believe that this selective effect comes from the difference in the practice volume of relevant actions in the sport. Other studies also confirmed that table tennis players responded faster to their dominant hand in mental hand rotation tasks, and gymnasts outperformed football players and nonathletes in mental rotation tasks involving multi-axial rotation [6, 10, 11]. Furthermore, Olympic diving involves multi-axial rotations and is characterized by significant height, time pressure, and spatial demands. Consequently, the spatial cognitive abilities required for diving have garnered increasing research interest and the advantage of divers were confirmed by some studies [12, 13].

As an critical individual difference variable, gender has been extensively investigated in the context of mental rotation research. Studies have shown that males’ spatial cognition ability is greater than that of females [14]. The researchers compared the mental rotation ability of 42 males and 42 females and found that gender was an effective factor in predicting mental rotation test scores; that is, males had stronger mental rotation ability than females. Fargier et al. [15] performed an MRt in 96 male and female undergraduate students, and the results showed that the log RT was 17% faster in the male subjects than in the female subjects. Influential factors may be individual physiological factors such as genetics, lateralization of brain function, sex hormones, and brain size. In addition, social and environmental factors and physical activity, such as level of socialization, early life experience, and sports training, may also affect gender differences in mental rotation [16, 17]. It is well known that many activities are generally considered “masculine”, such as mathematics, science and other subjects, and these subjects are included in the test of spatial ability. Therefore, if girls participate less in these masculine activities, the gender difference in mental rotation will be more significant; otherwise, the difference will be attenuated. One study investigated the recreational activities of preschool children in North America. The results showed that parents usually encouraged boys to play outdoor and more intense sports, while girls played more indoor and quiet games. The rich experience cultivated in activities such as tree climbing, football and computer games improved hand–eye coordination and enabled boys to perform better in spatial ability tests [17].

Nevertheless, the effect of motor skill learning on gender differences has not been extensively studied [18]. For male and female athletes who have been engaged in sports training for many years and who have similar experience, can gender differences be made up? The conclusions of the current studies are inconsistent. From the perspective of cognitive plasticity, the researchers believed that even if they both have sports experience, the gender gap still exists [8, 15, 19]. In contrast, some studies believe that female athletes can achieve greater improvements in activities; that is, gender differences can be compensated for by participation in physical activities [20]. Researchers believe that women can more significantly modify their visual search behavior during activity participation and thus perform better in the perceptual process or the encoding process of the mental rotation task [21] to increase their mental rotation score. The reason may be related to the type of stimulation. Jansen et al. [6] suggested that, according to the embodied theory, athletic girls perform better in mental rotation of familiar movements, but their performance on abstract mental rotation tasks that require certain transfers is quite different from that of male athletes. In summary, we hypothesize that the gender differences in mental rotation may disappear during the perceptual and encoding processes of the egocentric MR task.

In addition, in studies concerning mental rotation, far too little attention has been given to adolescent athletes. Gender differences in mental-rotation performance were significant for children under age 13 and increased during adolescence [22]. A recent meta-analysis of athletes’ mental rotation revealed that participants in all the studies examined were older than 17 years of age [18], possibly because sport expertise was developed over at least ten years of practice or because of delicate practices that begin in childhood [23]. In fact, some studies have demonstrated better mental rotation ability for children who regularly participate in sport training than for those who do not [24]. The mental rotation ability of two groups of girls aged 9–14 years was assessed, and the results indicated that the experimental group, which had participated in juggling training for 3 months, performed significantly faster than the control group, which had performed light strength training. There is a positive relationship between motor ability and accuracy on mental rotation tasks among primary school-aged and young children [25, 26]. Moreover, improvements in mental rotation performance in childhood are accompanied by increasing gender differences [27].

In summary, the present study aimed to elucidate the influence of sports experience on gender differences in mental rotation in young athletes and compared athletic boys’ and girls’ mental rotation ability under different transformations. This study hypothesized that (1) divers will outperform nonathletes in the objected-based and egocentric MR tasks. (2) boys will demonstrate better performance than girls in MR task with abstract images but not for the task with human body images.

## Materials and methods

### Ethical approval

This study was carried out ethically and approved by the Ethical Committee of Henan Sport University (No. 2022002). The research was conducted in accordance with the Declaration of Helsinki and all individuals in this manuscript provided written informed consent. Since the experimental subjects were adolescents, informed consent was obtained from both the subjects and their parents.

### Participants

A total of forty—four participants, comprising 22 divers (12 males and 10 females, with a mean age of 14.81 ± 2.50 years) and 22 nonathletes (12 males and 10 females, with a mean age of 13.18 ± 0.39 years), took part in the experiment. There was no significant difference in the age of the subjects between the two groups, *t* (42) = 1.069, *p* = 0.296,* d* = 0.32. The divers were affiliated with the Shanghai Diving Team, who had received 7—10 years of training and had approximately 30 h of training per week. They are all national-level athletes who had achieved top-eight in national competitions at least one time. Nonathlete subjects were students who had never participated in professional sports training. All participants in the study were junior high school students.

### Materials

**Fig. 1:**
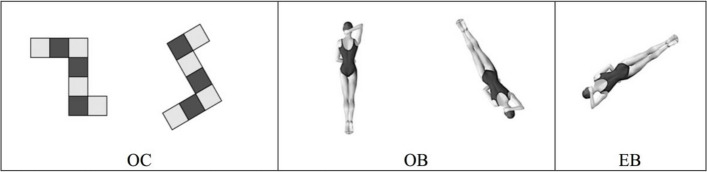
Experimental stimuli

The experiments were performed using the revised two-dimensional block image object and human body image. Three experimental conditions were included, representing two characterization methods: object-based transformations and egocentric transformations. (1) Object-based representation condition (i.e., Object-based cube-OC and Object-based body-OB): Two graphics were presented to the subjects each time. The left picture was the reference picture, and the right picture was the rotated picture (i.e., the two pictures were the same) or its mirror image (i.e., the two pictures were different). It is necessary to determine whether the two images are the same. The graphic is rotated clockwise in the horizontal plane, and the rotation angle is 0°, 30°, 60°, 90°, 120°, 150° or 180° (30° steps). (2) Egocentric representation conditions (egocentric body-EB): Only one figure of the human body was presented at each time point. The content and rotation angle of the figure were the same as those of the object representation conditions. The participants needed to determine which side of the person’s elbow was bent above the head in the figure (Fig. 1). The experimental task was designed with E-Prime 2.0 (Psychology Software Tools) and presented using a laptop computer with a 14-inch screen.

### Procedures

**Fig. 2:**
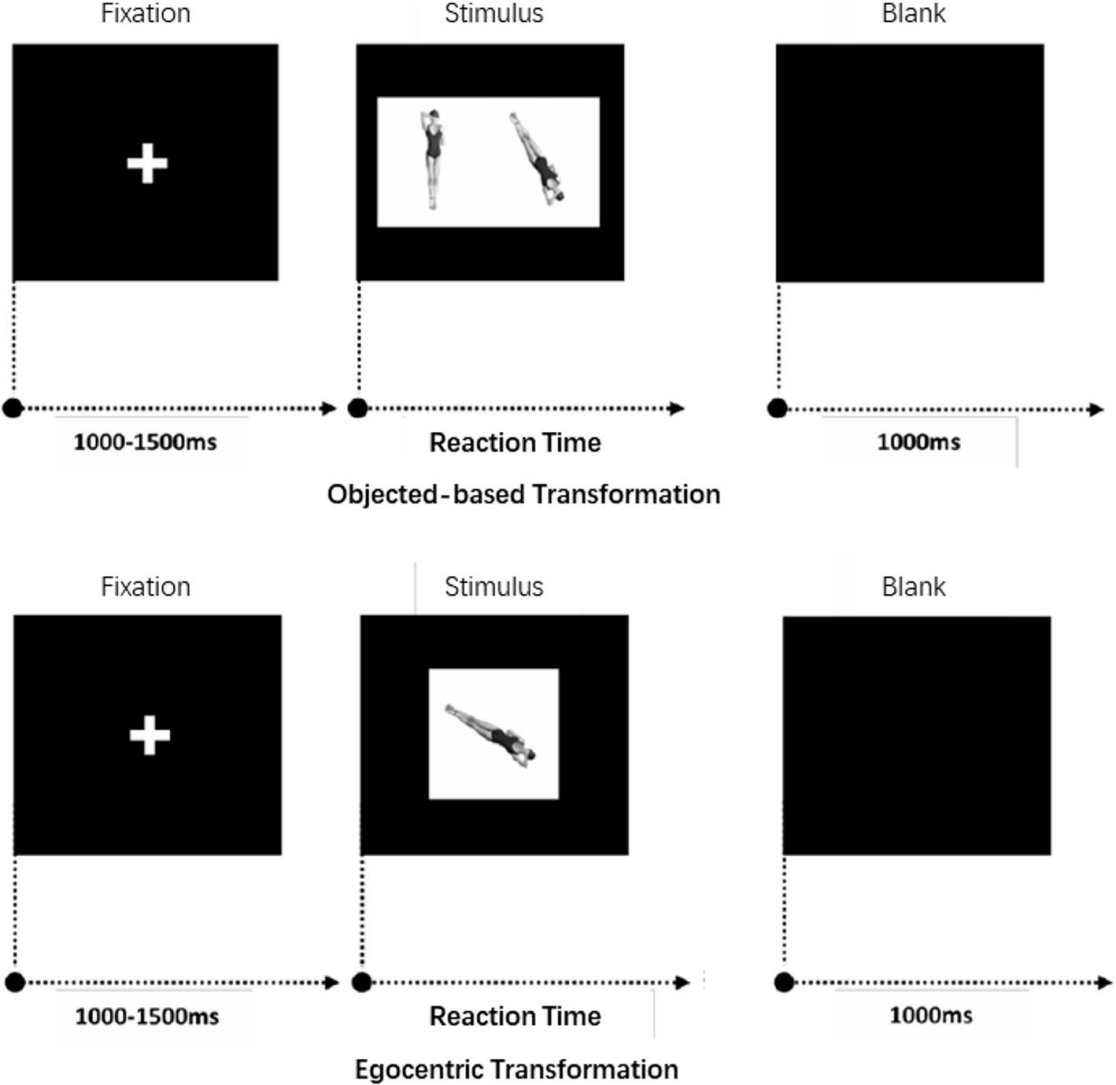
Example of human figure trials

The experiment was led by an experimenter and conducted one-on-one in a quiet conference room. After completing the personal information form, the subjects sat in front of the computer. After reading the experiment instructions, the participants were required to perform 20 practice trials with feedback. The formal experiment began with a correct response rate higher than 80%. In the object-based representation mental rotation task, the subjects needed to quickly and accurately determine whether the two images were the same or not and to respond by pressing the buttons with both hands, with the F key for the same and the J key for different. In the mental rotation task, the subjects needed to determine which side of the arm the person in the picture was bending over the head, with the F key for the left side and the J key for the right side. The experiment was divided into 2 random blocks according to the different characterization methods. A total of 112 trials were conducted, incorporating 7 rotation angles, 2 similarity-difference conditions, and 8 repetitions per condition. In each trial, the fixation point appeared for 1000–1500 ms, followed by the stimulus figure, which lasted until the participant pressed the key or disappeared after 3000 ms (Fig. 2). After a blank screen for 1000 ms, the next trial started. The duration of the experiment was approximately 45 min.

### Data statistics and analysis

Reaction time (RT), accuracy, perception stage reaction time and rotation speed were analyzed using multivariate analysis of variance (ANOVA). The between-group factors were group (athlete and nonathlete) and gender (boy, girl). Post hoc tests of the main effects and interactions were performed using the Bonferroni correction. Based on the data, subjects (2 athletes) who > *M* + 3*SD* accuracy < 0.85 were excluded. Prior to formal statistical analyses, the Shapiro–Wilk test was employed to assess the normality of the data distribution. The RTs of the two groups in the three conditions were transformed into a logarithm (*ln*) and showed a normal distribution (*z* < 1.097, *p* > 0.180, in all instances). Moreover, normal distributions were presented for the ERs of two groups in each condition (*z* < 1.345, *p* > 0.054, in all instances). To avoid the coverage of the experimental effects by multivariate interactions, the three experimental conditions were analyzed independently. According to the measurement of the mental rotation stage by Jansen and Just [21, 28], the RT when the stimulation material was not rotated (i.e., rotated 0°) was used as the evaluation index of the sensory stage, and the rotation speed was used as the performance of the rotation stage and it was the average of the ratio of the angle at each angle to the RT. The calculation formula is: rotation speed = $\left(\frac{30}{{RT}_{30}}+\frac{60}{{RT}_{60}}+\frac{90}{{RT}_{90}}+\frac{120}{R{T}_{120}}+\frac{150}{{RT}_{150}}+\frac{180}{{RT}_{180}}\right)\div 6\times 1000$, where the unit is represented in degree per second(◦/s). The calculation above was only carried out with the correct trials. All statistical analyses were conducted using IBM SPSS Statistics for Windows, Version 26.0.

## Results

### OC conditions

#### RT and accuracy

**Fig. 3:**
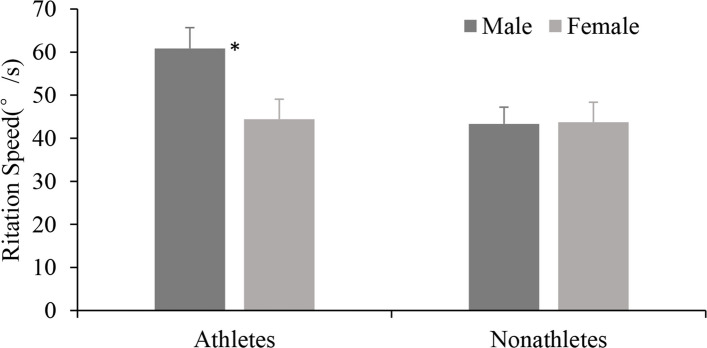
RT of divers and nonathletes of different genders under OC conditions (*M* ± *SE*)

After conducting the RT for the OC mental rotation test, the analysis showed that the main effect of group was significant [*F*(1, 40) = 6.477, *p* = 0.017, *ηp*^2^ = 0.193]. Athletes (2053 ± 266 ms) showed faster RT than nonathletes (2677 ± 299 ms). The main effect of gender [*F*(1, 40) = 0.375, *p* = 0.545, *ηp*^2^ = 0.014] and the interaction effect of the two [*F*(1, 40) = 0.578, *p* = 0.454, *ηp*^2^ = 0.021] were not significant; analysis of the accuracy revealed that the main effects for group [*F*(1, 40) = 0.089, *p* = 0.768, *ηp*^2^ = 0.003] and gender [*F*(1, 40) = 0.089, *p* = 0.768, *ηp*^2^ = 0.003] were not significant. These results showed that there was no difference in RT or accuracy between boys (2232 ± 313 ms; 0.89 ± 0.05) and girls (2497 ± 245 ms; 0.90 ± 0.06) in the mental rotation task for the OC condition. However, the RT of the athletic boys was relatively faster than that of the athletic girls (Fig. 3).

#### Stage performance

**Fig. 4:**
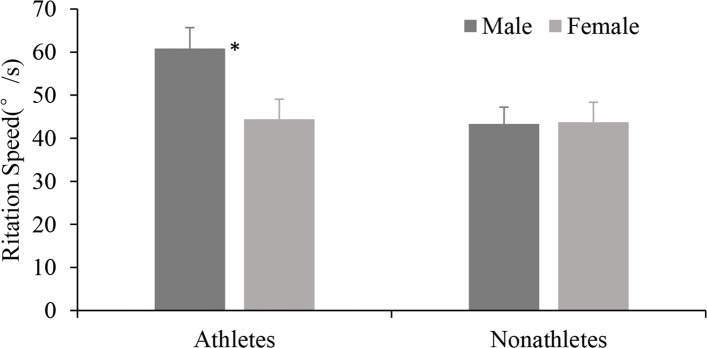
Rotation speeds of divers and nonathletes of different genders under OC conditions (*M* ± *SE*)

Analysis of the perceptual RT showed no significant differences between athletes and nonathletes [*F* (1, 40) = 0.597, *p* = 0.446, *ηp*^2^ = 0.022] or between boys and girls [*F* (1, 40) = 2.275, *p* = 0.110, *ηp*^2^ = 0.092]. However, the analysis of the rotation speed showed that the difference between the two groups [*F* (1, 40) = 3.430, *p* = 0.075, *ηp*^2^ = 0.113], showing faster rotation speed of the divers (51.01 ± 38°/s) than the nonathletes (40.55 ± 50°/s). The interaction between group and gender [*F* (1, 40) = 2.862, *p* = 0.099, *ηp*^2^ = 0.070] were marginally significant, but neither was the difference for gender [*F* (1, 40) = 2.229, *p* = 0.147, *ηp*^2^ = 0.076]. Post hoc analysis found that athletic boys (60.85 ± 44°/s) outperformed athletic girls (44.42 ± 32°/s) in the rotation phase (*p* < 0.05) (Fig. 4).

### OB conditions

#### RT and accuracy

**Fig. 5:**
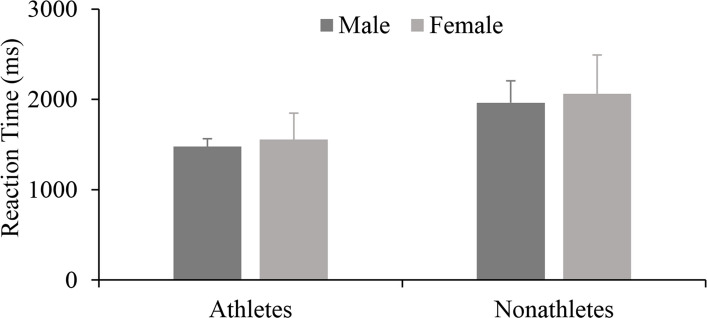
RT of divers and nonathletes of different genders under OB conditions (*M* ± *SE*)

**Fig. 6:**
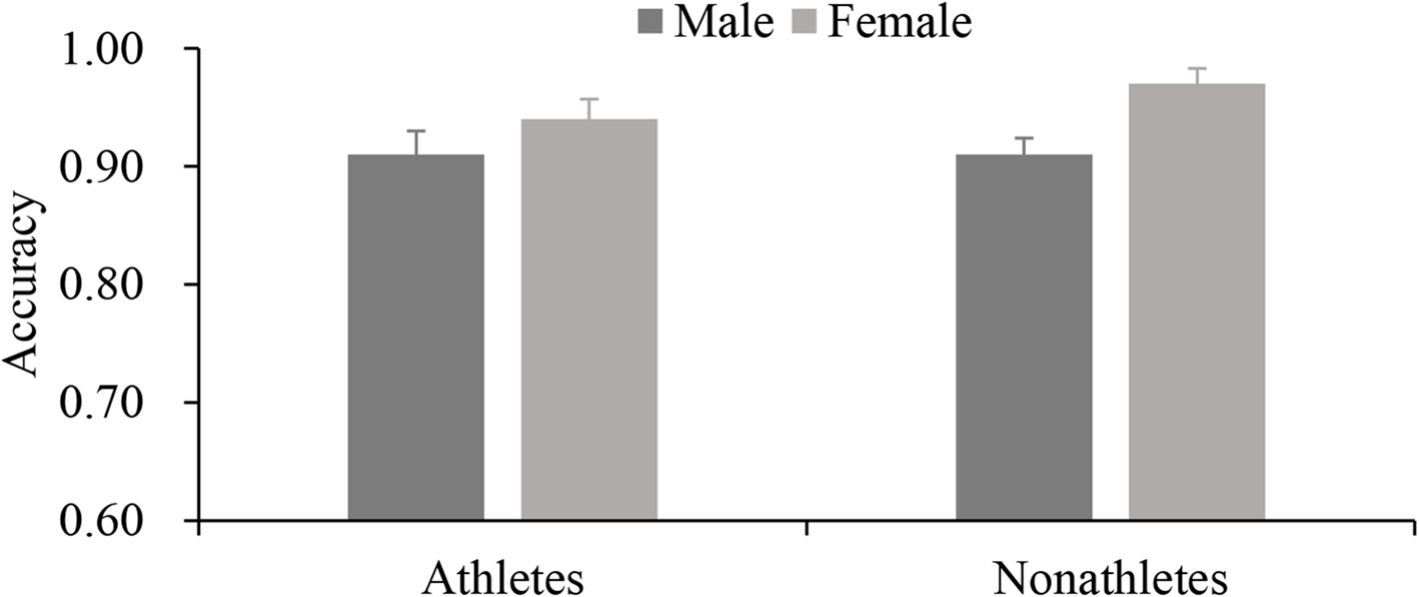
Accuracys for divers and nonathletes of different genders under OB conditions (*M* ± *SE*)

Analysis of RT showed that the main effect of group [*F* (1, 40) = 3.082, *p* = 0.087, *ηp*^2^ = 0.075], gender [*F* (1, 40) = 0.098, *p* = 0.756, *ηp*^2^ = 0.003] and their interaction [*F* (1,40) = 0.042, *p* = 0.839, *ηp*^2^ = 0.001] were not significant; analysis of accuracy showed that there was a gender difference [*F* (1,40) = 5.937, *p* = 0.019, *ηp*^2^ = 0.129]. However, the group difference was not significant [*F* (1, 40) = 0.655, *p* = 0.423, *ηp*^2^ = 0.016], and so was the interaction between the two variables [*F* (1, 40) = 0.919, *p* = 0.343, *ηp*^2^ = 0.022]. These results indicated that athletes (1516 ± 265 ms) responded faster than nonathletes (2012 ± 485 ms) in the task of object-based representation of human figures (Fig. 5). Inconsistent with the results in the OC condition, girls (0.96 ± 0.04) had a significantly greater accuracy than boys (0.91 ± 0.05) when mentally rotating object-based representations of human figures (*p* < 0.05) (Fig. 6).

#### Stage performance

Analysis of the perceptual stage revealed that, for both athlete and nonathlete groups [*F* (1, 40) = 2.118, *p* = 0.163, *ηp*^2^ = 0.105] and the boy and girl groups [*F* (1, 40) = 0.682, *p* = 0.420, *ηp*^2^ = 0.036], the difference was not significant. Subsequent analysis of the rotation stage also revealed no significant main effects for group [*F* (1, 40) = 0.909, *p* = 0.326, *ηp*^2^ = 0.023] or gender [*F* (1, 40) = 0.388, *p* = 0.541, *ηp*^2^ = 0.021]. This result indicated that there was no difference in performance between boys and girls in the perceptual and rotation phases of the mental rotation task for object-based body figures (Tables 1 and 2).

**Table 1:** RT at 0° for divers and nonathletes at the perception stage in the OB condition

Groups	Gender	Mean (ms)	Standard deviation (ms)
Athlete	Boy	994.75	210.74
	Girl	1120.69	718.73
	Total	1045.63	417.99
Nonathlete	Boy	1458.14	719.11
	Girl	1548.06	636.02
	Total	1491.75	673.73
Total	Boy	1244.72	522.63
	Girl	1334.38	674.18
	Total	1278.69	575.72

**Table 2:** Rotation speeds of divers and nonathletes at the rotation stage in the OB condition

Groups	Gender	Mean (°/s)	Standard deviation (°/s)
Athlete	Boy	58.11	18.57
	Girl	64.99	14.73
	Total	60.86	16.80
Nonathlete	Boy	54.94	18.46
	Girl	58.31	15.81
	Total	56.17	16.80
Total	Boy	56.40	17.80
	Girl	61.65	14.18
	Total	58.40	16.40

### EB conditions

#### RT and accuracy

**Fig. 7:**
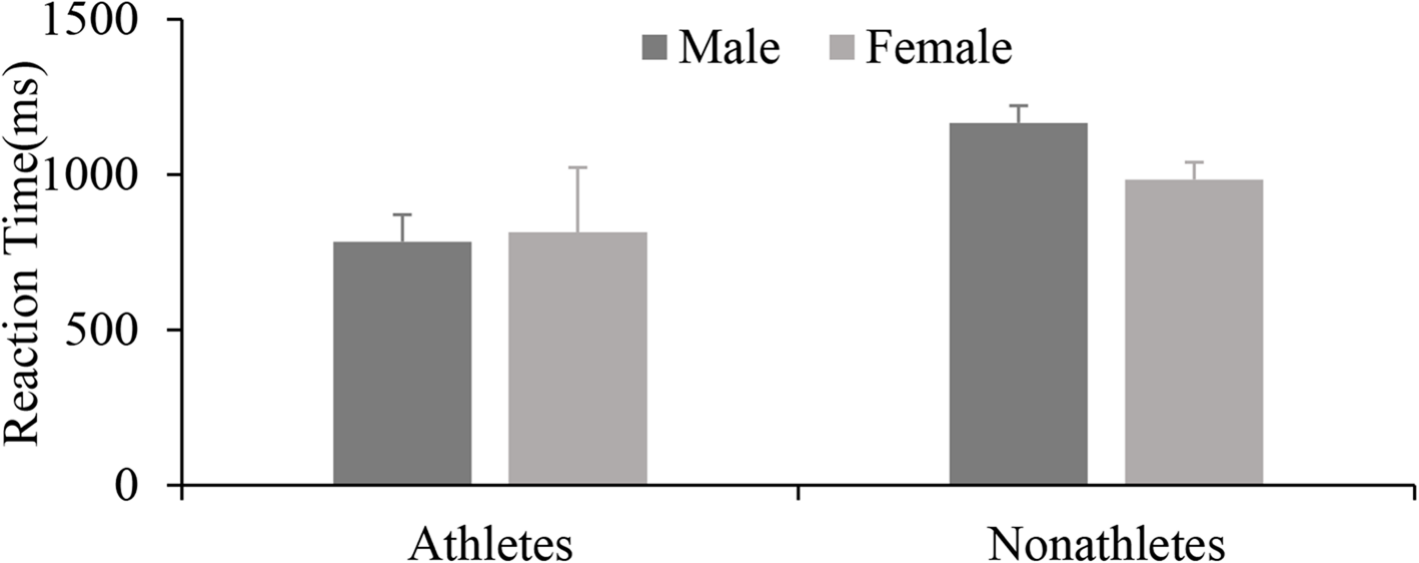
Response timing of divers and nonathletes of different genders according to egocentric representation (*M* ± *SE*)

Analysis of RT showed that the athlete group responded no significantly faster than the nonathlete group [*F* (1, 40) = 3.238, *p* = 0.080, *ηp*^2^ = 0.079], and the main effect of gender [*F* (1, 40) = 0.250, *p* = 0.620, *ηp*^2^ = 0.007] and the interaction were also not significant [*F* (1, 40) = 0.480, *p* = 0.493, *ηp*^2^ = 0.012]. Additionally, the accuracy results showed no main effects of group [*F* (1, 40) = 0.172, *p* = 0.681, *ηp*^2^ = 0.004] and gender [*F*(1,40) = 0.979, *p* = 0.329, *ηp*^2^ = 0.025] (Fig. 7).

#### Stage performance

The performance of the athletes was greater than that of the nonathletes in the perception stage [*F* (1, 40) = 4.719, *p* = 0.036, *ηp*^2^ = 0.110], but the main effects of gender [*F* (1, 40) = 0.074, *p* = 0.789, *ηp*^2^ = 0.004] and the interaction between the two [*F* (1, 40) = 0.010, *p* = 0.923, *ηp*^2^ = 0.001] were not significant, indicating that there was no significant difference in the perception speed between boys and girls. Similarly, in the rotation stage, the main effect of gender was not significant [*F* (1, 40) = 0.384, *p* = 0.539, *ηp*^2^ = 0.010], and the interaction between group and gender was not significant [*F* (1, 40) = 0.247, *p* = 0.622, *ηp*^2^ = 0.006]. However, there was a significant difference among individuals with different levels of sports experience [*F* (1, 40) = 7.863, *P* = 0.008, *ηp*^2^ = 0.171]; that is, the divers rotated faster. However, the rotation speeds of different genders during the process of egocentric rotation were almost the same (Tables 3 and 4).

**Table 3:** RT at 0° for divers and nonathletes at the perception stage in the EB condition

Groups	Gender	Mean (ms)	Standard deviation (ms)
Athlete	Boy	629.84	143.72
	Girl	608.16	75.20
	Total	620.94	120.64
Nonathlete	Boy	816.97	398.72
	Girl	796.94	208.03
	Total	809.68	329.35
Total	Boy	730.90	300.56
	Girl	702.55	176.50
	Total	719.50	257.97

**Table 4:** Rotation speeds of divers and nonathletes at the rotation stage in the EB condition

Groups	Gender	Mean (°/s)	Standard deviation (°/s)
Athlete	Boy	128.16	37.08
	Girl	118.42	17.43
	Total	124.91	31.64
Nonathlete	Boy	99.20	33.81
	Girl	97.80	14.10
	Total	98.69	27.83
Total	Boy	112.49	35.12
	Girl	108.13	17.95
	Total	110.83	30.03

Examples of three stimulation conditions (OC-object-based cube, OB-object-based body, EB-egocentric body). © QA International, 2017. All rights reserved

## Discussion

This study compared the overall and stage performance of object-based and egocentric mental rotation between divers and nonathletes of different genders. The RT, accuracy, RT in the perceptual stage and rotation speed were analyzed in detail. The results confirmed that the superior mental rotation ability of the athletes varied according to gender.

Hypothesis 1, that divers will outperform nonathletes in the objected-based and egocentric MR tasks, was verified. In particular, male athletes showed faster reaction times and rotation speeds during the OC task. Studies have confirmed that athletes’ simple RT and choice RT are faster than those of nonathletes [29–32] because athletes often need to quickly and accurately encode movement information at nerve centers, and relevant electrophysiological studies have shown that athletes have faster brain signal transmission speeds and shorter brain activity latencies [32]. Moreover, their better accuracy and shorter response times are correlated with increased activation in the bilateral intraparietal sulcus [33]. Moreover, athletic boys outperformed girls for the rotation speed. Voyer et al. conducted a meta-analysis of 286 relevant studies and showed significant male dominance in multiple mental rotation tests [18]. On the other hand, Heil and Jansen-Osmann [34] found that males mentally rotated more holistically because their rotational speed was independent of stimulus complexity. A study have showed that males and females differed in brain network connectivity for mental rotation [35]. Therefore, females rotated more analytically. From the perspective of embodied cognition [36], the advantage of athletic boys may be due to the embodied processing of mental rotation tasks into knowledge related to sports experience, thereby improving performance.

In the OB task, the present experiments investigated the influence of gender on the mental rotation ability of an object-based representation with human bodies. The results showed that the divers had faster RTs tendency in the task, which was consistent with the functional equivalence hypothesis [37], indicating that there was a high similarity between imagery and perception. To reveal the internal mechanism of the connection between body rotation and mental rotation, the present study selected the task stimulus of sport posture. Heinen [38] stressed the important value of sport stimulation images in mental rotation. The mental rotation performance of an athlete depends on whether the physical characteristics of the stimulation are consistent with the athlete’s own experience [6, 39]. In terms of accuracy in the OB task, there were significant gender differences, with a surprising result showing that girls had higher accuracy than boys. Jansen et al. confirmed that the gender difference exhibited by exercisers in the human figure task was significantly lower than that in abstract figures [6], which may lead athletic girls to perform better in mental rotation in familiar sports or movements. Paivio and Harshman used still picture learning and recall as homework tasks, respectively, and both found that females were dominant in appearance maintenance [40]. In an online experiment, 838 German-speaking participants solved four blocks of mental rotation trials with two or eight alternatives. Researchers did not observe meaningful sex differences at all [41]. Therefore, it seems that a key variable in predicting gender differences is whether the representations used in the completion of the task are static or dynamic. In this experiment, the task materials provided in the mental rotation object-based representation human figure task were all static pictures. Moreover, Rahe et al. [42] enrolled 189 undergraduate students to complete a mental rotation test with either male- or female-stereotyped objects. Significant gender differences appeared only when male-stereotyped objects were used as rotational material and not when female-stereotyped material was used. A study by Pietsch indicates that for women specific motor components seem to be more important for the development of mental rotation ability than gender-stereotype aspects [43]. The present study used the picture of young female athletes as task images, and the familiarity of the image for girls may cause greater accuracy, which confirmed Hypothesis 2.

In addition, the experiment investigated the effect of sports experience on the mental rotation ability of egocentric transformation. The results showed that divers performed better in terms of RT at the perception stage and rotation speed. In embodied theory, spatial transformation follows the perspective of “body imitation”. Action embodiment, as an embodiment method, refers to the consistency between the process of processing action perception, such as observation and imagination, and actual operation [32]. From this point of view, since mental rotation includes the imitation of action, this imitation may promote the retention of spatial information in action representation through action embodiment, thereby increasing the rotation speed [44]. Researchers attribute this result to a “selective effect”,that is, the greater the correlation between the task stimulus and the sports experience is, the greater the mental rotation score of the movement [10, 11, 20]. In addition, the results confirmed that boys and girls did not show any difference in overall or stage performance on the EB task. This may be related to the task characteristics. First, under the same circumstances as in the OB condition, girls have better responses because of the female stimuli. One study reported that boys were more familiar with male stimuli and girls were more familiar with female objects [45], thus, the familiarity of the rotational material could have an influence on performance. Second, the task characteristics responsible for gender differences in adults could be the difficulty of the test [46]. In the present study, the RTs in the EB condition for boys (976 ± 202 ms) and girls (899 ± 320 ms) were shorter than those in the other conditions, indicating that the task was easier to perform, which may have caused the discrepancy in gender. After all, the above findings confirmed hypothesis 1.

In view of the effect of age on the gender difference in mental rotation, although a study revealed that the gender difference in mental rotation may appear in the early years of individual development, boys and girls aged 10 years have shown differences in spatial ability [19]. The present study found superiority only for boys in object-based tasks but failed to detect any boy advantage in both object-based and egocentric tasks. It is possible that the nonathlete subjects were teenagers between the ages of 12 and 15 years who were receiving middle school education. Their daily learning involves more geometrical knowledge of figure transformation; therefore, boys and girls have similar spatial learning experiences for abstract images, and gender differences are not significant. In summary, an individual’s spatial cognitive ability may develop at different speeds in different age groups, and future research can explore the changes in mental rotation ability of individuals of different genders during the continuum of age development more from a vertical perspective, especially sports interventions.

## Conclusions

The present study utilized gender to assess the object-based and egocentric mental rotation abilities of adolescent divers and nonathletes. The mental rotation ability of adolescents was found to be significantly influenced by their mental rotation representation and motor expertise, which differed by gender. In the OC task, the perception speed of athletic boys was faster than that of athletic girls, showing the positive influence of sport training. Surprisingly, in the OB task, the accuracy for girls was significantly greater than that for boys. No gender difference was observed in the EB task, indicating that task characteristics such as transformation, difficulty and material may change the gender difference in mental rotation.

## Supplementary Information

Supplementary Material 1.

## Data Availability

All data generated or analyzed during this study are included in this published article and its supplementary information files.

## Abbreviations

OC: Objected-based cube
OB: Objected-based body
EB: Egocentric body
RT: Reaction time

## References

[1] Zacks JM, Ollinger JM, Sheridan MA, Tversky B. A parametric study of mental spatial transformations of bodies. Neuroimage. 2002;16(4):857–72. 10.1006/nimg.2002.1129. 12202075 10.1006/nimg.2002.1129

[2] Steggemann Y, Engbert K, Weigelt M. Selective effects of motor expertise in mental body rotation tasks: comparing object-based and perspective transformations. Brain Cogn. 2011;76(1):97–105. 10.1016/j.bandc.2011.02.013. 21429647 10.1016/j.bandc.2011.02.013

[3] Corballis MC. Recognition of disoriented shapes. Psychol Rev. 1988;95(1):115. 3281177 10.1037/0033-295x.95.1.115

[4] Heil M, Rolke B. Toward a chronopsychophysiology of mental rotation. Psychophysiology. 2002;39(4):414–22. 10.1017/S0048577202001105. 12212633 10.1017/S0048577202001105

[5] Shepard RN, Cooper LA. Mental images and their transformations. MIT Press; 1986.

[6] Jansen P, Lehmann J. Mental rotation performance in soccer players and gymnasts in an object-based mental rotation task. Adv Cogn Psychol. 2013;9(2):92–8. 10.2478/vl0053-008-0135-8. 23833695 10.2478/v10053-008-0135-8 PMC3700661

[7] Moreau D, Clerc J, Mansy-Dannay A, Guerrien A. Spatial ability and motor performance: assessing mental rotation processes in élite and novice athletes. Int J Sport Psychol. 2011;54(3):167–79.

[8] Schmidt M, Egger F, Kieliger M, Rubeli B, Schüler J. Gymnasts and orienteers display better mental rotation performance than non-athletes. J Individ Differ. 2015;37(1):1–7. 10.1027/1614-0001/a000180.

[9] Pietsch S, Jansen P. Different mental rotation performance in students of music, sport and education. Learn Individ Differ. 2012;22(1):159–63. 10.1016/j.lindif.2011.11.012.

[10] Habacha H, Lejeune-Poutrain L, Margas N, Molinaro C. Effects of the axis of rotation and primordially solicited limb of high level athletes in a mental rotation task. Hum Mov Sci. 2014;37:58–68. 10.1016/j.humov.2014.06.002. 25064695 10.1016/j.humov.2014.06.002

[11] Habacha H, Molinaro C, Tabben M, Lejeune-Poutrain L. Implementation of specific motor expertise during a mental rotation task of hands. Exp Brain Res. 2014;232(11):3465–73. 10.1007/s00221-014-4029-3. 25027791 10.1007/s00221-014-4029-3

[12] Feng T, Li Y. The time course of event-related brain potentials in athletes’ mental rotation with different spatial transformations. Front Behav Neurosci. 2021;15:675446. 10.3389/fnbeh.2021.675446. 34211377 10.3389/fnbeh.2021.675446 PMC8239182

[13] Feng T, Zhang F, Liang M, Liu J, Wei Y. Sport training of axial rotation affects spatial ability: evidence from behavior and fNIRS. Heliyon. 2024;10(23):e39340. 10.1016/j.heliyon.2024.e39340. 39698083 10.1016/j.heliyon.2024.e39340 PMC11652840

[14] Hegarty M, Waller DA. Individual Differences in Spatial Abilities. In P. Shah (Ed.) & A. Miyake, The Cambridge Handbook of Visuospatial Thinking Cambridge University Press. 2005;121–69. 10.1017/CBO9780511610448.005.

[15] Fargier P, Champely S, Massarelli R, Ammary L, Hoyek N. Modelling response time in a mental rotation task by gender, physical activity, and task features. Sci Rep. 2022;12(1):15559. 10.1038/s41598-022-19054-2. 36114235 10.1038/s41598-022-19054-2 PMC9481519

[16] Peters M, Manning JT, Reimers S. The effects of sex, sexual orientation, and digit ratio (2D: 4D) on mental rotation performance. Arch Sex Behav. 2007;36(2):251–60. 17394056 10.1007/s10508-006-9166-8

[17] Siegel-Hinson RI, McKeever WF. Hemispheric specialisation, spatial activity experience, and sex differences on tests of mental rotation ability. Laterality. 2002;7(1):59–74. 10.1080/13576500143000078. 15513188 10.1080/13576500143000078

[18] Voyer D, Jansen P. Motor expertise and performance in spatial tasks: a meta-analysis. Hum Mov Sci. 2017;54:110–24. 10.1016/j.humov.2017.04.004. 28437638 10.1016/j.humov.2017.04.004

[19] Pietsch S, Jansen P. The relationship between coordination skill and mental rotation ability. In Spatial Cognition VIII (pp. 173–181). Springer. 2012.

[20] Habacha H, Molinaro C, Dosseville F. Effects of gender, imagery ability, and sports practice on the performance of a mental rotation task. Am J Psychol. 2014;127(3):313–23. 25588273 10.5406/amerjpsyc.127.3.0313

[21] Jansen P, Lehmann J, Van Doren J. Mental rotation performance in male soccer players. PLoS ONE. 2012;7(10):e48620–e48620. 10.1371/journal.pone.0048620. 23119073 10.1371/journal.pone.0048620 PMC3484043

[22] Voyer D. Magnitude of sex differences in spatial abilities. Psychol Bull. 1995;117(2):250–70. 7724690 10.1037/0033-2909.117.2.250

[23] Ericsson KA, Krampe RT, Tesch-Römer C. The role of deliberate practice in the acquisition of expert performance. Psychol Rev. 1993;100(3):363–406.

[24] Jansen P, Lange L, Heil M. The influence of juggling on mental rotation performance in children. Biomed Hum Kinet. 2011;3(2):223–9. 10.2478/v10101-011-0005-6.

[25] Jansen P, Heil M. The relation between motor development and mental rotation ability in 5- to 6-year-old children. Eur J Dev Sci. 2010;4(1):66–74.

[26] Jansen P, Kellner J. The role of rotational hand movements and general motor ability in children’s mental rotation performance. Front Psychol. 2015;6:1–11. 10.3389/fpsyg.2015.00984. 26236262 10.3389/fpsyg.2015.00984 PMC4503890

[27] Levine SC, Foley A, Lourenco S, Ehrlich S, Ratliff K. Sex differences in spatial cognition: advancingthe conversation. Wiley Interdisciplinary Reviews:Cognitive Science. 2016;7(2):127–55. 26825049 10.1002/wcs.1380

[28] Just MA, Carpenter PA. Cognitive coordinate systems: accounts of mental rotation and individual differences in spatial ability. Psychol Rev. 1985;92(2):137–72. 3887449

[29] Heirani A, Vazinitaher A, Soori Z, Rahmani M. Relationship between choice reaction time and expertise in team and individual sports: a gender differences approach. Aust J Basic Appl Sci. 2012;6(8):344–8.

[30] Kioumourtzoglou E, Kourtessis T, Michalopoulou M, Derri V. Differences in several perceptual abilities between experts and novices in basketball, volleyball and water-polo. Percept Mot Skills. 1998;86(1):899–912. 9656285 10.2466/pms.1998.86.3.899

[31] Piras A, Lobietti R, Squatrito S. Response time, visual search strategy, and anticipatory skills in volleyball players. J Ophthalmol. 2014;2014(4):189268. 24876946 10.1155/2014/189268 PMC4021845

[32] Zwierko T, Osinski W, Lubinski W, Czepita D, Florkiewicz B. Speed of visual sensorimotor processes and conductivity of visual pathway in volleyball players. J Hum Kinet. 2010;23(1):21–7.

[33] Semrud-Clikeman M, Fine JG, Bledsoe J, Zhu DC. Gender differences in brain activation on a mental rotation task. Int J Neurosci. 2012;122(10):590–7. 10.3109/00207454.2012.693999. 22651549 10.3109/00207454.2012.693999

[34] Heil M, Jansen-Osmann P. Sex differences in mental rotation with polygons of different complexity: do men utilize holistic processes whereas women prefer piecemeal ones? Q J Exp Psychol (Hove). 2008;61(5):683–9. 18421643 10.1080/17470210701822967

[35] Zhang K, Fang H, Li Z, Ren T, Li B-m, Wang C. Sex differences in large-scale brain network connectivity for mental rotation performance. Neuroimage. 2024;298:120807. 10.1016/j.neuroimage.2024.120807. 39179012 10.1016/j.neuroimage.2024.120807

[36] Wexler M, Kosslyn SM, Berthoz A. Motor processes in mental rotation. Cognition. 1998;68(1):77–94. 9775517 10.1016/s0010-0277(98)00032-8

[37] Riečanský I, Jagla F. Linking performance with brain potentials: mental rotation-related negativity revisited. Neuropsychologia. 2008;46(13):3069–73. 18639565 10.1016/j.neuropsychologia.2008.06.016

[38] Heinen T. Does the athletes’ body shape the athletes’ mind? A few ideas on athletes’ mental rotation performance. Commentary on Jansen and Lehmann. Adv Cogn Psychol. 2013;9(2):99–101. 10.2478/v10053-008-0136-7. 23833696 10.2478/v10053-008-0136-7 PMC3700736

[39] de Lange FP, Helmich RC, Toni I. Posture influences motor imagery: an fMRI study. Neuroimage. 2006;33(2):609–17. 16959501 10.1016/j.neuroimage.2006.07.017

[40] Paivio A, Harshman R. Factor analysis of a questionnaire on imagery and verbal habits and skills. Psychology/Revue canadienne de psychologie. 1983;37(4):461–83.

[41] Jost L, Jansen P. The influence of the design of mental rotation trials on performance and possible differences between sexes: a theoretical review and experimental investigation. Q J Exp Psychol. 2024;77(6):1250–71. 10.1177/17470218231200127 PMC11103899 37644655

[42] Rahe M, Ruthsatz V, Quaiser-Pohl, C. Influence of the stimulus material on gender differences in a mental-rotation test. Psychol Res. 2021;85(8):2892–9. 33237389 10.1007/s00426-020-01450-w

[43] Pietsch S, Jansen P. Motor affordance or gender-stereotyped nature of physical activity – what is more important for the mental rotation performance of female athletes? J Cogn Psychol. 2021;33(5):568–80. 10.1080/20445911.2021.1931242.

[44] Amorim M-A, Isableu B, Jarraya M. Embodied spatial transformations: “body analogy” for the mental rotation of objects. J Exp Psychol Gen. 2006;135(3):327–47. 10.1037/0096-3445.135.3.327. 16846268 10.1037/0096-3445.135.3.327

[45] Ruthsatz V, Neuburger S, Rahe M, Jansen P, Quaiser-Pohl C. The gender effect in 3D-Mental-rotation performance with familiar and gender-stereotyped objects – a study with elementary school children. J Cognitive Psychol. 2017;29(6):717–30.

[46] Collins DW, Kimura D. A large sex difference ona two-dimensional mental rotation task. Behav Neurosci. 1997;111(4):845–9. 9267662 10.1037//0735-7044.111.4.845

