# Supplementary material for: The influence of motor expertise on gender difference in adolescents’ object-based and egocentric mental rotation ability
Source: BMC Psychol. 2026 Feb 16;14:422. doi: 10.1186/s40359-026-04099-z (PMC13032329; doi:10.1186/s40359-026-04099-z)
Supplement: Supplementary file 1 — Supplementary Material 1. [file 40359_2026_4099_MOESM1_ESM.zip › Material/ethics.pdf]

伦理委员会审批表

编号：[郑体]伦审字（2022002）号

研究方案名称及编号	运动经验对运动员空间认知的影响		
试验产品名称		研究类型	1☐ 2☐ 3☐ 4☐ 5☐<br>6☐ 7☐ 8☐ 9■10■11■
申请单位（教研室）	教育心理	项目负责人	冯甜
申办者	冯甜	CRO（如适用）	
参加单位	郑州大学体育学院		
审查文件	申请表		

投票	同意	做必要修正后同意	做必要修改后重审	不同意	终止或暂停已批准的试验
	10 票	0 票	0 票	0 票	0 票

1. 评审意见和建议：
   研究方案符合伦理要求，同意实施。

2. 根据以上意见和建议，委员会对该方案的审查决定如下：
   ■同意☐做必要修正后同意☐做必要修改后重审☐不同意☐终止或暂停已批准的试验

3. 该研究进行过程中将接受伦理委员会的持续审查/审查频率？
   ☐是☐1 个月☐6 个月☐12 个月
   ■否

主任委员/副主任委员签名：[illegible]

日期：2022-2-10

备注：1=药物临床试验Ⅰ期；2=药物临床试验Ⅱ期；3=药物临床试验Ⅲ期；4=药物临床试验Ⅳ期；5=国际多中心药物临床试验；6=其他类别药物临床试验；7=医疗器械临床试验；8=医疗新技术的临床研究；9=运动训练研究；10=动物实验；11=科研课题

Ethics committee approval form

No：202202

<table>
<tr><td>Study protocol and serial number</td><td colspan="3">Effect of exercise experience on spatial cognition in athletes</td></tr>
<tr><td>Test product name</td><td></td><td>Research type</td><td>1☐ 2☐ 3☐ 4☐ 5☐
6☐ 7☐ 8☐ 9■10■11■</td></tr>
<tr><td>Application unit (Teaching and Research Section)</td><td>Psychology of sports</td><td>Project leader</td><td>Tian Feng</td></tr>
<tr><td>applicant</td><td>Tian Feng</td><td>CRO (If applicable)</td><td></td></tr>
<tr><td>Affiliation</td><td colspan="3">Physical Education College of Zhengzhou University</td></tr>
<tr><td>file</td><td colspan="3">approval form</td></tr>
</table>

<table>
<tr><td rowspan="2">vote</td><td>Agree</td><td>Agree after revision</td><td>apply again after revision</td><td>Disagree</td><td>Terminate or suspend</td></tr>
<tr><td>10</td><td>0</td><td>0</td><td>0</td><td>0</td></tr>
</table>

1. Review opinions and suggestions:
   The study protocol met the ethical requirements and agreed to the implementation.

2. Based on the above comments and recommendations, the Committee reviews the protocol as follows:
   ■ Agree with ☐ to make the necessary amendments and agree with ☐ to make the necessary modifications and review ☐ disagree with ☐ to terminate or suspend the approved trial

3. Frequency of ongoing ethics committee review / review during the study?
   ☐ Is ☐ 1 month ☐ 6 months ☐ 12 months ■ deny

Sign:

日期：2022-2-10

1= phase I drug clinical trial; 2= drug clinical trial; 3= drug clinical trial; 4= drug clinical trial; 5= international multi-center drug clinical trial; 6= clinical trial of other types of drugs; 7= medical device clinical trial; 8= clinical research of new medical technology; 9= exercise training research; 10= animal experiment; 11= scientific research project
