# Supplementary material for: The influence of motor expertise on gender difference in adolescents’ object-based and egocentric mental rotation ability
Source: BMC Psychol. 2026 Feb 16;14:422. doi: 10.1186/s40359-026-04099-z (PMC13032329; doi:10.1186/s40359-026-04099-z)
Supplement: Supplementary file 1 — Supplementary Material 1. [file 40359_2026_4099_MOESM1_ESM.zip › Material/ethic english.pdf]

Ethics committee approval form

No：202202

<table>
<tr><td>Study protocol and serial number</td><td colspan="6">Effect of exercise experience on spatial cognition in athletes</td></tr>
<tr><td>Test product name</td><td colspan="2"></td><td colspan="2">Research type</td><td colspan="2">1☐ 2☐ 3☐ 4☐ 5☐
6☐ 7☐ 8☐ 9■10■11■</td></tr>
<tr><td>Application unit (Teaching and Research Section)</td><td colspan="2">Psychology of sports</td><td colspan="2">Project leader</td><td colspan="2">Tian Feng</td></tr>
<tr><td>applicant</td><td colspan="2">Tian Feng</td><td colspan="2">CRO (If applicable)</td><td colspan="2"></td></tr>
<tr><td>Affiliation</td><td colspan="6">Physical Education College of Zhengzhou University</td></tr>
<tr><td>file</td><td colspan="6">approval form</td></tr>
<tr><td rowspan="2">vote</td><td>Agree</td><td>Agree after revision</td><td>apply again after revision</td><td>Disagree</td><td colspan="2">Terminate or suspend</td></tr>
<tr><td>10</td><td>0</td><td>0</td><td>0</td><td colspan="2">0</td></tr>
</table>

1．Review opinions and suggestions:
The study protocol met the ethical requirements and agreed to the implementation.

2．Based on the above comments and recommendations, the Committee reviews the protocol as follows:
■ Agree with ☐ to make the necessary amendments and agree with ☐ to make the necessary modifications and review ☐ disagree with ☐ to terminate or suspend the approved trial

3．Frequency of ongoing ethics committee review / review during the study?
☐ Is ☐ 1 month ☐ 6 months ☐ 12 months ■ deny

Sign:

日期：2022-2-10

1= phase I drug clinical trial; 2= drug clinical trial; 3= drug clinical trial; 4= drug clinical trial; 5= international multi-center drug clinical trial; 6= clinical trial of other types of drugs; 7= medical device clinical trial; 8= clinical research of new medical technology; 9= exercise training research; 10= animal experiment; 11= scientific research project
